# *Salvia miltiorrhiza* extract protects white matter and the hippocampus from damage induced by chronic cerebral hypoperfusion in rats

**DOI:** 10.1186/s12906-015-0943-6

**Published:** 2015-11-23

**Authors:** Min-Soo Kim, Ji Hye Bang, Jun Lee, Hyeon Woo Kim, Sang Hyun Sung, Jung-Soo Han, Won Kyung Jeon

**Affiliations:** Korea Institute of Oriental Medicine, 1672 Yuseongdae-ro, Yuseong-gu, Daejeon 305-811 Republic of Korea; Department of Internal Medicine, School of Medicine, Keimyung University, 1095 Dalgubeoldae-ro, Dalseo-gu, Daegu 704-701 Republic of Korea; College of Pharmacy and Institute of Pharmaceutical Research, Seoul National University, 1 Gwanak-ro, Gwanak-gu, Seoul 151-742 Republic of Korea; Department of Biological Sciences, Konkuk University, 120 Neungdong-ro, Gwangjin-gu, Seoul 143-701 Republic of Korea; Korean Medicine Life Science, University of Science & Technology, Daejeon, 34054 Republic of Korea

**Keywords:** *Salvia miltiorrhiza*, Chronic cerebral hypoperfusion, Myelin basic protein, Toll-like receptor

## Abstract

**Background:**

*Salvia miltiorrhiza* (SM), an herbal plant, is traditionally used in the treatment of cardiovascular and cerebrovascular diseases in Asian countries. SM has multiple biological effects including anti-inflammatory activity. The present study is aimed at investigating the effects of SM extract in rats with chronic cerebral hypoperfusion.

**Methods:**

Chronic cerebral hypoperfusion was induced in male Wistar rats by permanent bilateral common carotid artery occlusion (BCCAo). The rats were divided into 3 groups: sham-control, BCCAo treated with vehicle, and BCCAo treated with SM extract. Vehicle or SM extract (200 mg/kg) were administered daily by oral gavage beginning on day 21 after BCCAo and continuing to day 42. Immunohistochemical analyses were used to measure Iba-1-positive microglia and myelin basic protein (MBP) in white matter and hippocampal tissue. In addition, the expression levels of proinflammatory cytokines, including TNF-α, IL-1β, and IL-6, and the toll-like receptor (TLR) pathway in the hippocampus, were analyzed by western blot.

**Results:**

Administration of SM extract attenuated the activation of microglial cells in the white matter and hippocampus after BCCAo. SM extract also prevented neuroinflammation after BCCAo by reducing hippocampal levels of TNF-α, IL-1β, and IL-6, and increasing the reduced levels of MBP in the white matter and hippocampus. Further, the administration of SM extract alleviated the up-regulation of hippocampal TLR4 and myeloid differentiation primary response gene 88 (MyD88) in rats with chronic BCCAo.

**Conclusions:**

Our findings suggest that SM may be a promising therapeutic candidate in vascular dementia because of its protective effects against damage to the white matter and hippocampus after BCCAo.

## Background

Vascular dementia (VaD), caused by changes in the blood supply of the brain, is the second most common form of dementia [1]. Neural dysfunction and cognitive impairment occur in response to chronic cerebral hypoperfusion, which is observed in patients with VaD [2]. The effects of chronic cerebral hypoperfusion can be studied in a widely accepted animal model using permanent occlusion of the bilateral common carotid artery (BCCAo) in rats [3, 4]. BCCAo-induced damage to white matter and the hippocampus has been reported in many studies [5, 6]. The development of pathological features is associated with the activation of inflammatory cytokines such as interleukin-1 beta (IL-1β), IL-6, and tumor necrosis factor alpha (TNF-α) [4, 7, 8]. Furthermore, the toll-like receptor (TLR) pathway has an important function during cerebral hypoperfusion as well as mitogen-activated protein kinase (MAPK) signaling [4, 7, 9, 10]. For example, TLR4-deficient mice are protected against the inflammatory response following cerebral ischemia [11].

*Salvia miltiorrhiza* (Fam. *Labiatae,* SM), a traditional herbal plant, is commonly used to treat the symptoms of cardiovascular and cerebrovascular diseases in Korea, China, Japan, and other Asian countries [12–15]. The medium lethal dose of SM is 40–80 g/kg, which implies that SM has low acute toxicity compared to the toxicity of most of the other herbal medicines [16]. The beneficial effects of SM have been demonstrated on ischemia-induced symptoms including cellular damage and low blood flow [14]. In addition, several research groups reported the effects of SM on inflammatory responses. Treatment of human vein endothelial cells with SM caused a significant decrease of IL-6 and IL-8 [17]. Oral gavage with polysaccharide from SM reduced TNF-α and IL-1β levels in mouse models of liver injury [18]. Thus, SM has the potential to improve the pathology of cerebrovascular diseases through anti-inflammatory effects.

Although several studies revealed that SM has effects on cerebral ischemia including stroke, no study regarding its potential impact on chronic cerebral hypoperfusion has been reported. Therefore, in order to examine the effect of SM on damaged brain tissue following chronic cerebral hypoperfusion, SM extract was administered by oral gavage to rats with BCCAo. These animals were then examined to determine whether SM extract treatments ameliorated BCCAo-induced brain damage. Specifically, the effects of SM extract on the activation of microglia and myelin injury were examined in the white matter and hippocampus after chronic BCCAo. In addition, levels of pro-inflammatory cytokines, and the expression of proteins associated with the TLR pathway, were evaluated in the hippocampus.

## Methods

### Animals

Forty-two 12-week-old male Wistar rats were used in the chronic BCCAo experiments (Charles River Co., Gapyung, South Korea). For two weeks prior to beginning the experiment, the rats were housed in a vivarium at the Korea Institute of Oriental Medicine (KIOM) under controlled temperature (22 ± 1 °C) and humidity (55 ± 10 %) with a 12-h light/dark cycle. Food and water were given ad libitum to all rats in this experiment. All experimental procedures described in this report were approved by the Institutional Animal Care and Use Committee of the KIOM (permit number: 13–088).

### Preparation of SM extracts

SM, produced in China, was obtained in 2011 from a commercial supplier (Kwangmyung-Dang, Ulsan, Korea). It was identified by the Herbal Quality Control Team and deposited at the Creative Research Laboratory of the KIOM. Dried SM was pulverized and extracted in hot water for 3 h at room temperature by using an ultrasound-assisted extractor (Daegu Haany University, Daegu, Korea). The extract was filtered and then concentrated under vacuum using a rotary evaporator (500 g, 33.2 % yield).

### Ultra-performance liquid chromatography-quadrupole time-of-flight mass spectrometry (UPLC-QTOF/MS) analyses of SM extracts

**Fig. 1:**
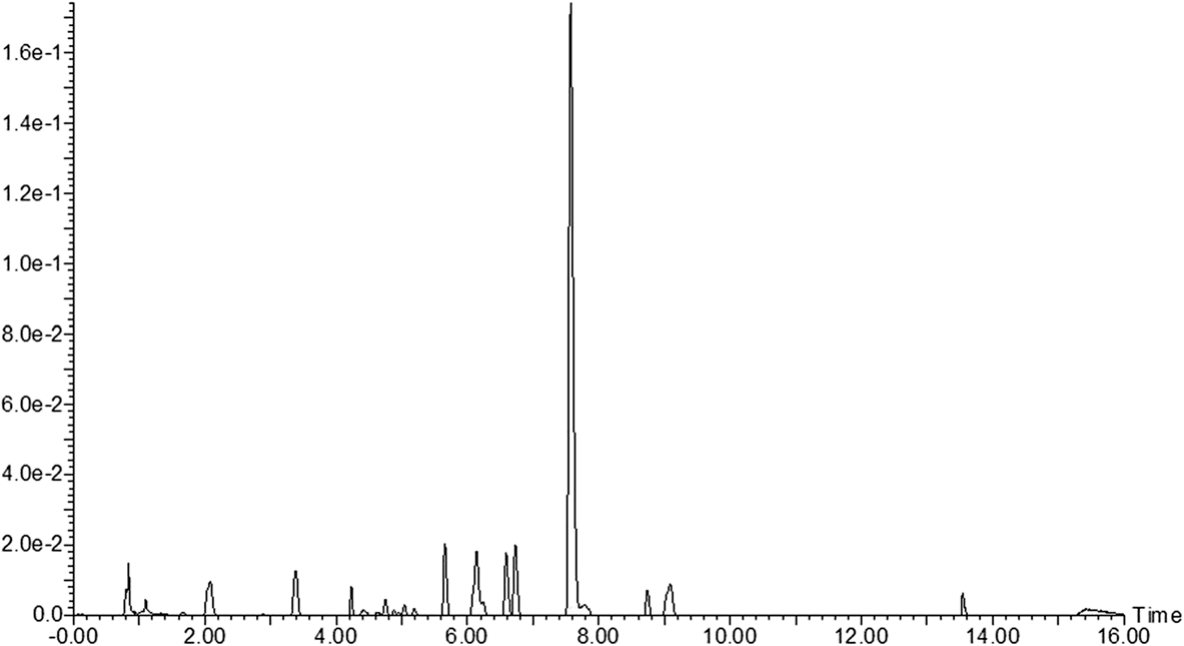
UPLC-DAD chromatogram of an SM extract. 1. Salvianic acid A; 2. protocatechuic aldehyde; 3. caffeic acid; 4. salvianolic acid G; 5. salvianolic acid E/L; 6. rosmarinic acid; 7. salvianolic acid A; 8. salvianolic acid B; 9. salvianolic acid E/L

**Fig. 2:**
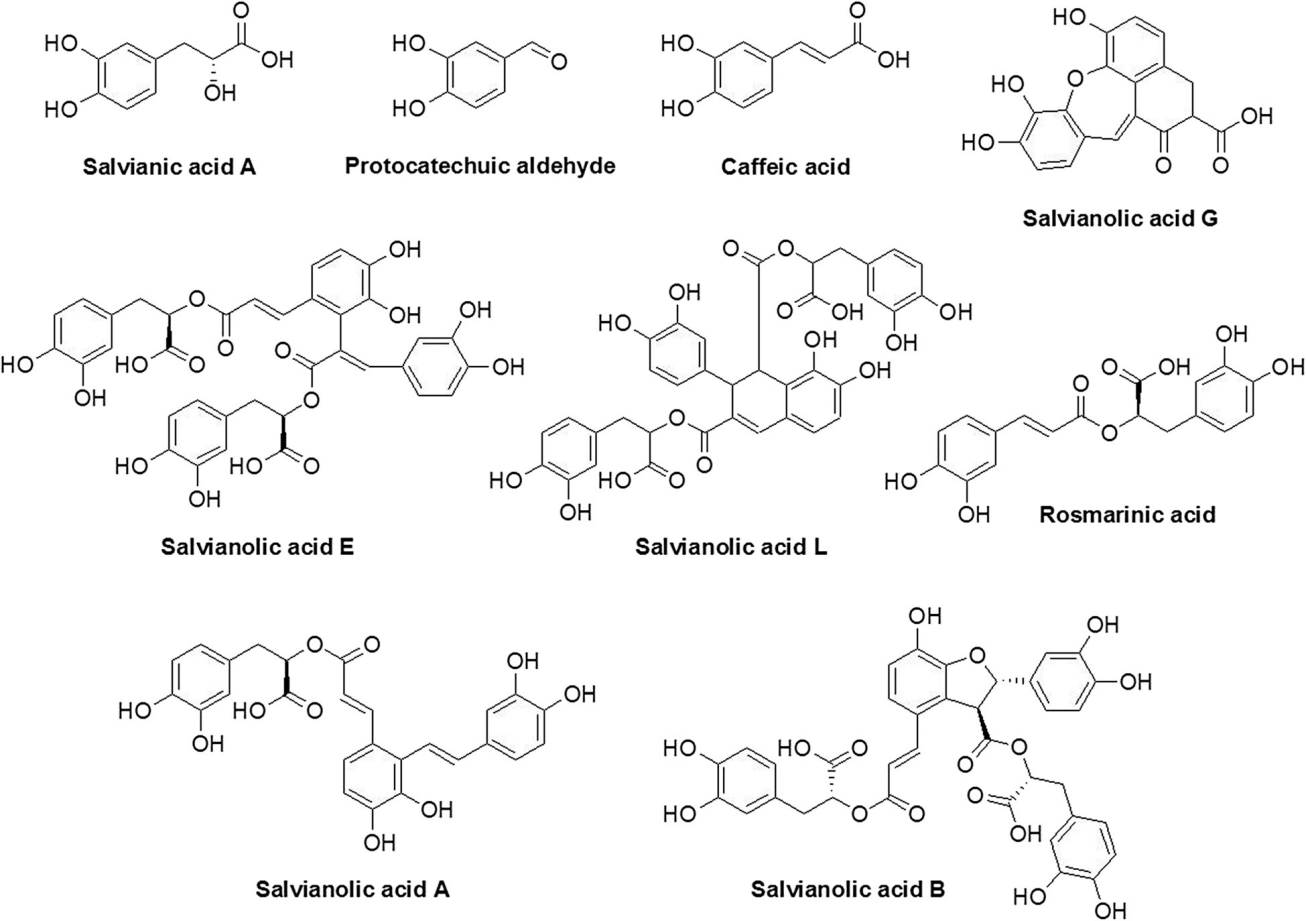
Chemical structures of constituents in the SM extract

The constituents of the SM extract were analyzed and identified by the UPLC-QTOF/MS technique (Figs. 1, 2 and Table 1). UPLC-QTOF/MS data were acquired from an Acquity UPLC system (Waters Co., Milford, MA, USA) that consisted of a binary solvent delivery system, auto sampler, and photo diode array detector. A Waters Aquity UPLC BEH C18 (150 mm × 2.1 mm, 1.7 μm) column was used. The mobile phases were 20 mM formic acid in H_2_O (A) and acetonitrile (B), with the following gradient (A to B): 5–11.5 % (0–3 min), 11.5–20 % (3–3.1 min), 20–25 % (3.1-10 min), and 25–100 % (10–14 min). The flow rate was set at 300 μL/min and the injection volume was 2.0 μL. The temperatures of the auto sampler and column oven were maintained at 15 and 40 °C, respectively. The chromatogram was recorded at 280 nm.

**Table 1:** Chromatograms and high resolution mass data of constituents in the SM extract

Peak NO.	Retention time (min)	HR-mass [M-H]^−^	Tolerance (ppm)	λ_max_ (nm)	Formula	Compound
1	2.13	197.0446	−2.0	200, 279	C_9_H_10_O_5_	Salvianic acid A
2	3.44	137.0238	−0.7	233, 279, 311	C_7_H_6_O_3_	Protocatechuic aldehyde
3	4.30	179.0335	−5.0	245, 325	C_9_H_8_O_4_	Caffeic acid
4	5.72	339.0489	−4.7	252, 286, 306	C_18_H_12_O_7_	Salvianolic acid G
5	6.18	717.1433	−3.2	247, 330	C_36_H_30_O_16_	Salvianolic acid E/L
6	6.65	359.0761	−1.7	244, 330	C_18_H_16_O_8_	Rosmarinic acid
7	6.78	493.1120	−3.0	253, 308	C_26_H_22_O_10_	Salvianolic acid A
8	7.63	717.1453	−0.4	228, 309	C_36_H_30_O_16_	Salvianolic acid B
9	8.80	717.1446	−0.7	253, 286, 306	C_36_H_30_O_16_	Salvianolic acid E/L

Mass spectrometry experiments were performed using a Xevo G2 QTOF mass spectrometer (Waters MS Technologies, Manchester, UK), which was connected to the UPLC system through an electrospray ionization interface. Electrospray ionization conditions were set as follows: negative ion mode, capillary voltage, 2.5 kV’ cone voltage, 40 V; source temperature, 120 °C; desolvation, 350 °C; cone gas flow, 50 L/h; and desolvation gas flow, 800 L/h. The ion acquisition rate was 0.2 s. Data were centroided during acquisition using an independent reference lock-mass ion via the LockSpray™ interface to ensure accuracy and precision. Leucine encephalin (m/z 554.2615 in negative mode) was used at 200 pg/μL with an infusion flow rate of 5 μL/min.

### HPLC analysis

The quantitation of major compound, salvianolic acid B, in SM extract was performed by HPLC analysis. The HPLC-DAD system consisting of a chromatographic pump (P680, Dionex, Idstein, Germany), an automated sample injector (ASI-100, Dionex), and a thermostatted column compartment (TCC-100, Dionex) equipped with an UVD 340U detector (Dionex). The chromatogram of HPLC system was recorded by using Dionex’s Chromeleon™ Chromatography Data System. The column used in this work was a Capcell Pak C-18 column (150 mm × 4.6 mm, I.D., 5 μm, Shiseido Co., Ltd., Japan.). The column temperature was set to 30 °C. The mobile phase consisted of (A) 0.1 % formic acid in water and (B) 100 % acetonitrile at a flow rate of 1.0 mL/min. The gradient condition was as follows: 0–5 min, 10 %–30 % B; 5–25 min, 30 %–40 % B; 25–30 min, 40 %–100 % B.

### Calibration curve and HPLC quantitation analysis

The calibration curve of salvianolic acid B (98 % purity, Shanghai Sunny Biotech Co., Ltd., China) showed good linearity (*R*^2^ = 0.9995) within the linear range (0.0125–2 mg/mL) and the equation is *y* = 64.242*x*-0.2516 (*y*, peak area; *x*, concentration of analyte). The sample solution (10 mg/mL) was prepared for HPLC quantitation analysis. The filtered solution of sample (10 μl) was injected into the instrument. The sample was prepared in triplicate. Peaks in the chromatograms were identified by comparing the retention time and UV spectra with those of the standard. The content of analyte was calculated from calibration curve. The content of salvianolic acid B in SM extract is 12.6 ± 0.03 mg/g drug.

### Surgery and SM administration

Rats were anesthetized with 5 % isoflurane in a mixture of 30 % oxygen/70 % nitrogen. BCCAo surgery was modified based on previous reports [10, 19]. A midline incision was performed to expose both common carotid arteries that were then double-ligated tightly using silk sutures 4–0. Control rats were subjected to a sham operation in which they underwent the same procedure without BCCAo. During the surgical procedure, body temperature was maintained at 37.0 ± 0.5 °C and all efforts were made to minimize pain. We confirmed reduced glucose utilization following BCCAo which imply established hypoperfusion (data not shown).

**Fig. 3:**
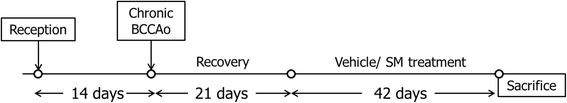
Experimental design

Rats were divided into three groups: a sham-operated group with oral administration of saline (*n* = 12); a BCCAo group with oral administration of saline (*n* = 15); and a BCCAo group with oral administration of SM extract 200 mg/kg (*n* = 15). The dose of SM extract was determined based on prescreening results. Oral gavage treatments with saline or SM extract were initiated on day 21 after BCCAo or sham surgery and continued daily until day 42 (see Fig. 3). During the experiments, two rats died in both the saline and the SM treatment groups owing to stress related to surgery and long-term oral feeding. The rats displayed no toxicity with respect to changes in general behavior and mortality during the oral treatments.

### Western blot analyses

Tissue preparation and western blot were carried according to a previously reported study with modifications [20]. Hippocampal tissue was homogenized in cold RIPA buffer containing 25 mM Tris HCl pH 7.6, 150 mM NaCl, 1 % NP-40, 1 % sodium deoxycholate, 0.1 % SDS (Thermo Scientific, Waltham, MA, USA), and protease and phosphatase inhibitor cocktail solution (GenDEPOT, Barker, TX, USA), and centrifuged at 20,000 × g for 30 min at 4 °C. The supernatants were harvested and stored at −70 °C. The protein concentration of each supernatant was determined using the BCA assay (Thermo Scientific). Equal amounts of protein (40 μg) were separated via SDS-PAGE and transferred to a PVDF membrane that was incubated with a primary antibody against TNF-α (Santa Cruz Biotechnologies, Santa Cruz, CA, USA), IL-1β (Millipore Corporation, Billerica, MA, USA), IL-6 (Abcam, San Francisco, CA, USA), TLR4 (Santa Cruz), or myeloid differentiation factor 88 (MyD88, Santa Cruz). Glyceraldehyde-3-phosphate dehydrogenase (GAPDH, Santa Cruz) was used as the internal control. A goat anti-rabbit horseradish peroxidase-conjugated secondary Ab (Cell Signaling, Danvers, MA, USA) was then added and detection was done using an ECL system (Thermo Scientific) with a Lumino Image Analyzer (Las-4000; Fujifilm, Tokyo, Japan). Densitometry was performed for specific markers normalized to GAPDH using Multi Gauge software (Fujifilm).

### Immunohistochemistry

Immunohistochemical analysis was performed as described previously [3]. The brain was post-fixed in 4 % paraformaldehyde for 7 days, cryoprotected in phosphate-buffered saline containing 30 % sucrose for 14 days at 4 °C, and then stored at −70 °C. Cryosections of the brain (40 μm) were incubated for 16 h at 4 °C with ionized calcium binding adaptor molecule-1 (Iba-1, Wako, Tokyo, Japan), myelin basic protein (MBP, Abcam), and NeuN (Millipore) in phosphate buffered saline containing 3 % casein and 0.1 % Triton-X 100, and then incubated with a goat anti-rabbit antibody (Cell Signaling Technology). Finally, the stained sections were treated with a Vector SG substrate kit and a Vector DAB kit (Vector Laboratories, Burlingame, CA, USA) for peroxidase-mediated staining, and mounted onto resin-coated slides. The dried sections on the slides were coverslipped using Permount reagent (Fisher Scientific, Pittsburgh, PA, USA). The stained sections were examined under light microscopy (Bx 51; Olympus, Japan) and the number of Iba-1-positive microglial cells was assessed in regions (0.03 mm^2^) of white matter (the corpus callosum, fimbria, and optic tract) and the hippocampus (CA1, CA3, and DG). The cell count data are presented as an average of Iba-1 positive cell in each region. In addition, MBP level was measured in the hippocampus, fornix, medial septum, fimbria, and corpus callosum. The optical density of the MBP was measured using Metamorph analysis software (Molecular Devices, Sunnyvale, CA, USA). A minimum of three different Iba-1- and MBP-stained sections per animal were averaged for quantification.

### Statistical analyses

Differences between groups were considered significant at *p* < 0.05. One-way ANOVA with a post hoc test (Least Significant Difference test) was performed to determine the effects of SM extract administration on alterations in the number of MBP and Iba-1-positive cells, as well as the expression levels of TNF-α, IL-1β, IL-6, TLR4, and MyD88 induced by chronic BCCAo. All data are presented as means ± standard deviation (S.D.).

## Results

### SM extract attenuated microglial proliferation in the white matter and hippocampus of rats with chronic BCCAo

**Fig. 4:**
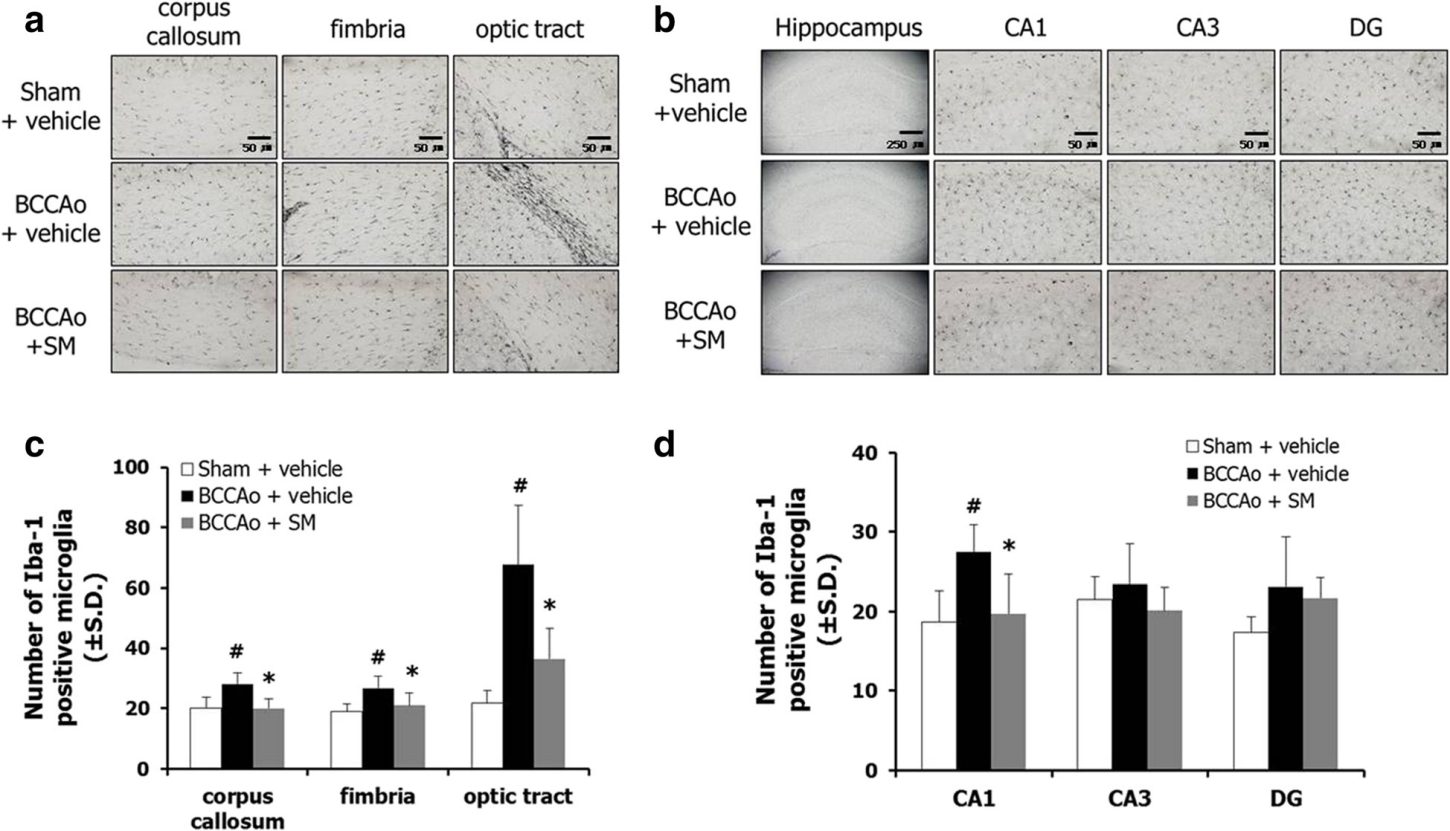
The effects of SM extract on chronic BCCAo-induced microglial proliferation in white matter and the hippocampus. **a** Photomicrographs illustrating Iba-1-positive cells in the corpus callosum, fimbria, and optic tract**. b** Photomicrographs illustrating Iba-1-positive cells in the hippocampal subregions. **c** Quantitative analysis of the number of Iba-1-positive cells in the corpus callosum, fimbria, and optic tract. **d** Quantitative analysis of the number of Iba-1-positive cells in the hippocampal subregions**. S**ham-control group (*n* = 6), BCCAo + vehicle group (*n* = 7), and BCCAo + SM group (*n* = 7). Data are presented as means ± S.D. ##*p* < 0.01 compared with sham-control; *p < 0.05 and **p < 0.01 compared with BCCAo + vehicle. Original magnification 40× for whole hippocampus and 200× for the other regions. Scale bar on entire hippocampus represents 250 μm and the other subregions represent 50 μm

Chronic cerebral hypoperfusion proliferates inflammatory cells within the brain, including microglia [6, 21]. Therefore, to examine the effects of SM extract treatment on neuroinflammation, the number of Iba-1-positive microglia in the major regions of white matter (corpus callosum, fimbria, and optic tract) and hippocampal formations (CA1, CA3, and DG) were measured (Fig. 4a, b). ANOVA revealed significant group effects of Iba-1 positive microglia in the corpus callosum, fimbria, optic tract, and hippocampal CA1 formation (F(2,17) ≥ 7.263, *p* < 0.005). An ANOVA for the hippocampal CA3 and DG formations showed no group effects. Post-hoc analyses revealed that the number of Iba-1-positive microglia in the vehicle-treated BCCAo rats was significantly higher than the sham-control rats in the corpus callosum, fimbria, optic tract, and hippocampal CA1 formation (Fig. 4c, d). The number of Iba-1 positive microglia was less in the BCCAo rats treated with SM extract than in the vehicle-treated BCCAo rats in the above-mentioned areas (Fig. 4c, d).

Based upon NeuN immunostaining, no clear neuronal cell death was observed in the hippocampal formation after chronic BCCAo. Furthermore, the administration of SM extract did not affect neuronal cell death (data not shown). These results suggest that SM extract administration mitigated chronic BCCAo-induced proliferation of microglia in the white matter and hippocampus.

### SM extract attenuated the increased expression of proinflammatory cytokines in the hippocampus of rats with chronic BCCAo

**Fig. 5:**
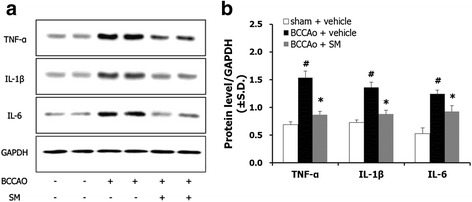
The effects of SM extract on proinflammatory cytokines in the hippocampus induced by chronic BCCAo. **a** Representative western blots of TNF-α, IL-1β, and IL-6. **b** Levels of TNF-α, IL-1β, and IL-6 in the sham-control group (*n* = 6), BCCAo + vehicle group (*n* = 6), and BCCAo + SM group (*n* = 6). Data are presented as means ± S.D. ##*p* < 0.01 compared with sham-control; *p < 0.05 and **p < 0.01 compared with BCCAo + vehicle

Activated microglial cells release proinflammatory cytokines [22]. To evaluate the effects of SM extract on the up-regulated proinflammatory cytokines induced by chronic BCCAo, western blotting was used to measure hippocampal levels of TNF-α, IL-1β, and IL-6. ANOVA showed significant group effects with all of these proteins (F(2,15) ≥ 10.063, *p* < 0.005). Post-hoc analyses revealed that chronic BCCAo increased the levels of these cytokines in the hippocampus compared to sham-control animals (Fig. 5). Increases in proinflammatory cytokines by chronic BCCAo were not observed in the chronic BCCAo rats treated with SM extract (Fig. 5). These results indicate that SM extract administration yields improved functional outcomes by reducing the proinflammatory cytokines induced by chronic BCCAo.

### SM extract up-regulated the reduced MBP in the white matter and hippocampus after chronic BCCAo

**Fig. 6:**
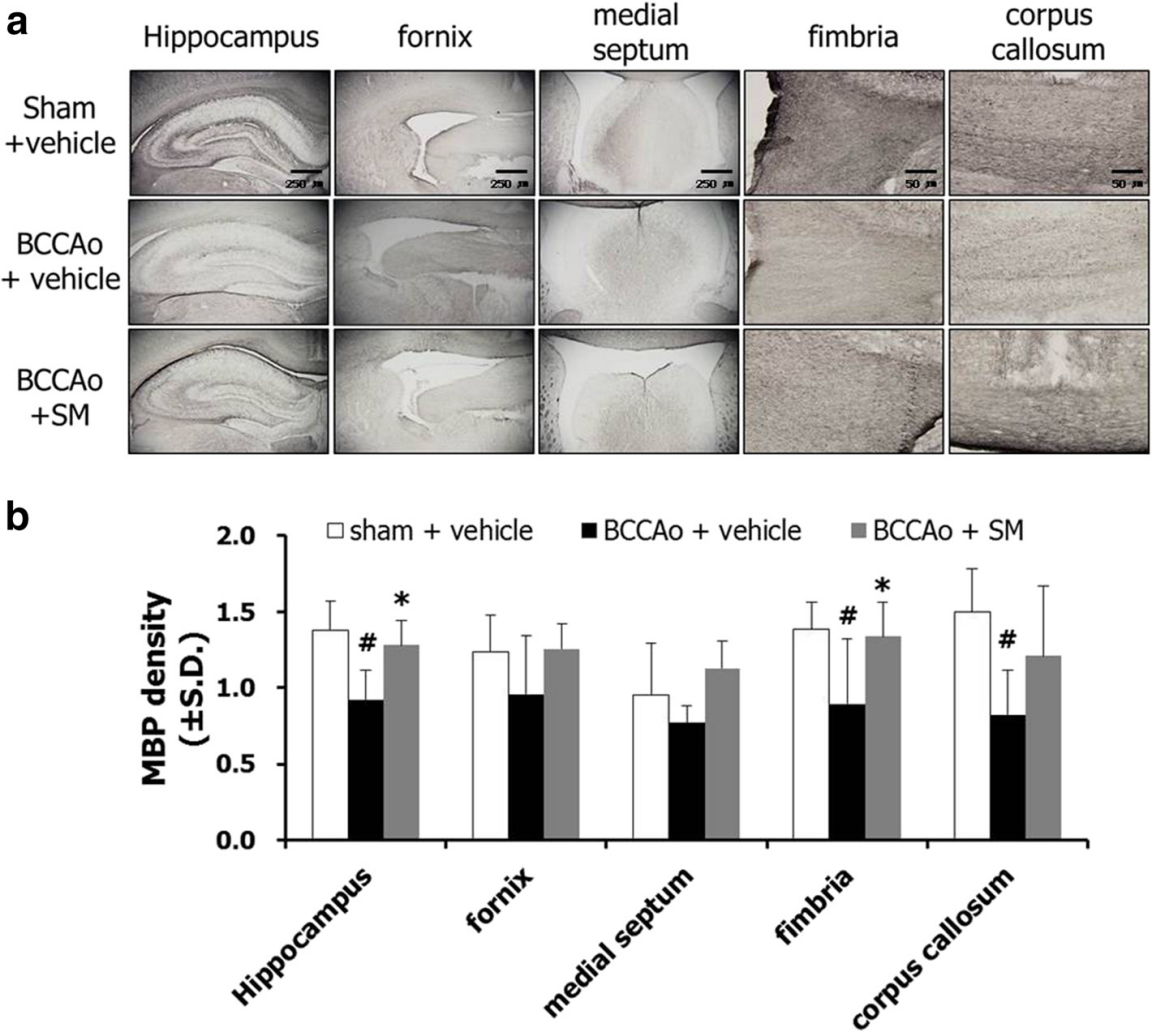
The effects of SM extract on the chronic BCCAo-induced reduction of MBP in the white matter and hippocampus. **a** Photomicrographs of MBP positive cells in the hippocampus, fornix, medial septum, fimbria, and corpus callosum. **b** Levels of MBP in the sham-control group (*n* = 6), BCCAo + vehicle group (*n* = 7), and BCCAo + SM group (*n* = 7). MBP density is expressed as arbitrary units. Data are presented as means ± S.D. #*p* < 0.05 and ##*p* < 0.01 compared with sham-control; *p < 0.05 and **p < 0.01 compared with BCCAo + vehicle. Original magnification 40× for the hippocampus and 200× for the other regions. Scale bar on hippocampus, fornix, and medial septum represent 250 μm and fimbria and corpus callosum represent 50 μm

In earlier studies, microglial cells were suggested to contribute to myelin impairment through the activation of proinflammatory cytokines including TNF-α [23, 24]. Disruption of myelinated fibers caused by chronic cerebral hypoperfusion has been also observed [7, 25, 26]. In order to investigate the effects of SM extract on the breakdown of myelin sheaths induced by chronic BCCAo, MBP, a component of the myelin sheath, was measured in the hippocampus, fornix, medial septum, fimbria, and corpus callosum. ANOVA revealed significant group effects in the hippocampus, medial septum, fimbria, and corpus callosum (F(2,17) ≥ 4.293, *p* < 0.05). However, there were no group effects detected in the fornix. Post-hoc analyses showed that the level of MBP in the chronic BCCAo rats treated with vehicle was significantly decreased in the hippocampus, fimbria, and corpus callosum (Fig. 6). SM extract administration prevented the loss of MBP following chronic BCCAo in the hippocampus and fimbria (Fig. 6). These data indicate that SM extract reversed the decrease in MBP in the white matter and hippocampus caused by chronic BCCAo.

### SM extract mitigated activation of the TLR4 pathway in the hippocampus after chronic BCCAo

**Fig. 7:**
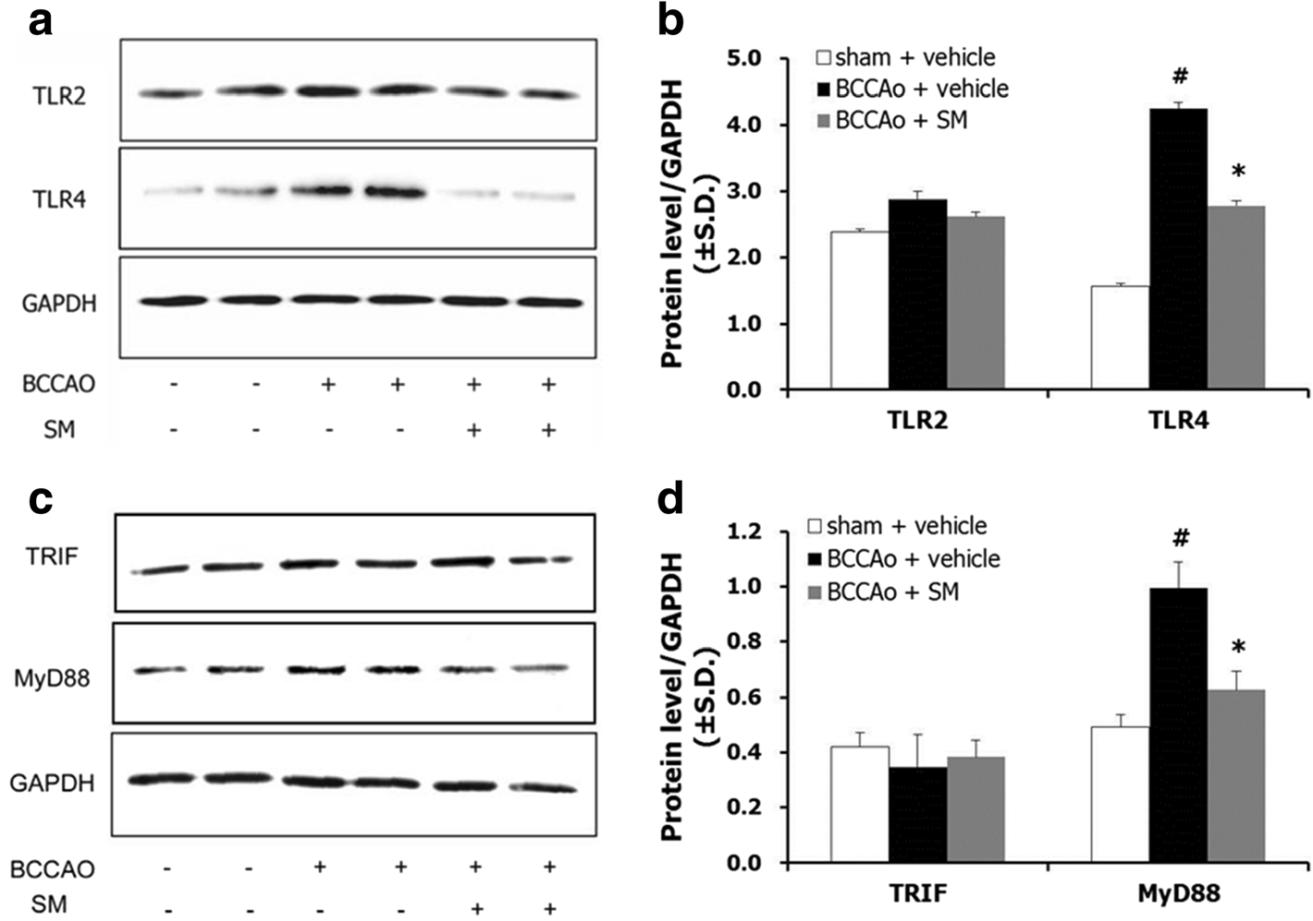
The effects of SM extract on TLR-related proteins in the hippocampus after chronic BCCAo. Representative western blots of (**a**) TLR2 and TLR4, and (**c**) TRIF and MyD88. Levels of (**b**) TLR-related and (**d**) TRIF and MyD88 proteins in the sham-control group (*n* = 4), BCCAo + vehicle group (*n* = 4), and BCCAo + SM group (*n* = 4). Data are presented as means ± S.D. ##*p* < 0.01 compared with sham-control; *p < 0.05 and **p < 0.01 compared with BCCAo + vehicle

Several studies have reported that glial cells express TLRs and that their activation has detrimental effects in cerebral ischemia [27–29]. Therefore, western blotting was used to measure hippocampal levels of TLR2 and TLR4. ANOVA showed significant group effects in TLR4 but not TLR2 expression (F(2,9) = 16.618, *p* < 0.001). Subsequent post-hoc analyses revealed that the administration of SM extract decreased the increased TLR4 seen after chronic BCCAo (Fig. 7a, b).

To determine whether the effects of chronic BCCAo occurred through MyD88-dependent pathways, hippocampal levels of Toll/IL-1R domain–containing adaptor-inducing IFN-β (TRIF), and MyD88, in the downstream pathway of TLR4 [30], were measured. ANOVA revealed significant group effects in MyD88 but not in TRIF expression (F(2,9) = 24.458, *p* < 0.001). This suggests that SM extract regulates the MyD88-dependent TLR pathway. Post-hoc analyses showed that SM extract administration reduced the increase of MyD88 caused by BCCAo (Fig. 7c, d). Taken together, these results suggest that the administration of SM extract regulated changes in the TLR4/MyD88 pathway caused by chronic BCCAo.

## Discussion

SM, also known as “Danshen,” is a traditional Asian medicine. It has been commonly used for the treatment of cardiovascular and cerebrovascular diseases [12, 31, 32]. The biological effects of SM and its extract have been clarified in the last 20 years, providing evidence for anti-inflammatory [33], anti-proliferative [34], and anti-apoptotic effects [35]. Safety monitoring of SM also has been conducted. Treatment of patients with hypertension and diabetes with SM seems to have little effect on the metabolism of lipids and glucose, blood pressure, and heart rate [36, 37]. These results indicate that SM could be a useful and safe natural drug candidate.

Chemical analysis of SM extract by UPLC showed that it contains some phenolic compounds, such as rosmarinic acid, caffeic acid, and salvianolic acid A and B [37, 38]. These phenolic acids may have antioxidant activity against peroxidative damage. Traumatic brain injury induced in mice leads to cognitive impairment and neuroinflammation. Treatment of these animals with salvianolic acid B, one of the active constituents of SM, restored spatial memory and inhibited the expression of proinflammatory cytokines [39]. Other studies have revealed that salvianolic acid B has antioxidant effects, protects against ischemia in rats, and restores memory functions affected by cerebral transient ischemia in mice [40, 41]. In addition, rosmarinic acid, which is one of the bioactive compounds of *Echium amoneum*, inhibits the expression of cyclooxygenase-2 in transient BCCAo in rats [42]. Caffeic acid and its derivatives found in *Coriandrum sativum* have antioxidant properties following 30 min of BCCAo in rats [43, 44]. These results imply that these phenolic compounds could provide protection against the chronic BCCAo that occurs when cerebral blood flow is reduced, similar to transient BCCAo. Accordingly, it is possible that SM exerts its beneficial effects through the activity of its phenolic acid constituents.

A large body of evidence indicates that SM can ameliorate brain injury. For example, intraperitoneal injection of SM in ischemia-reperfusion injured rats reduced the region of cerebral infarction [45]. However, no previous study has examined the effect of SM on damage to the white matter and hippocampus induced by chronic cerebral hypoperfusion. The present study explored the beneficial effect of SM extract on chronic BCCAo.

Evidence indicates that the proliferation of glia, such as astrocytes and microglia, occurred in response to both transient and chronic cerebral hypoperfusion [46, 47]. Increased numbers of glial cells contribute to the release of inflammatory cytokines that are capable of causing brain damage [46]. Thus, inhibition of glial proliferation, and consequently proinflammatory cytokines, may be critical in recovering from chronic BCCAo.

We demonstrated previously that the chronic BCCAo rat model is suitable for examining the effectiveness of natural medicines for VaD [7, 10]. Daily treatment with SM extract or vehicle was started 21 days after BCCAo surgery based on the results of our previous report, which showed that inflammatory molecules peaked at this time [3]. The present study showed that SM extract inhibited chronic BCCAo-induced microglia cell proliferation in the white matter and hippocampus. Additionally, down-regulation of increased hippocampal TNF-α, IL-1β, and IL-6 was observed in the chronic BCCAo rats treated with SM extract. These findings suggest that SM may possess protective effects on chronic cerebral hypoperfusion via inhibition of the inflammatory response.

We also identified the effect of SM extract on the expression of MBP after BCCAo in the hippocampus and white matter. MBP is associated with stabilization of the myelin sheath, a key element of white matter [26]. Demyelination induced by chronic cerebral hypoperfusion can damage not only white matter but also gray matter including the hippocampus [48, 49]. Specifically, it has been hypothesized that proinflammatory molecules released by microglia accelerate myelin degradation [24, 50]. Confirming previous reports [7], the expression of MBP was down-regulated in response to chronic BCCAo in the hippocampus and white matter. However, the decrease in MBP was not observed in rats with chronic BCCAo given SM extract. These findings suggest that SM may suppress the breakdown of MBP following chronic cerebral hypoperfusion. In addition, these data imply that SM might be beneficial in the treatment of other demyelinating diseases including multiple sclerosis [51]. Further studies are needed to determine the mechanisms underlying the effects of SM on myelin disruption.

TLR signaling plays a role in the immune response. TLRs are expressed constitutively within glial cells [52]. Among TLRs, TLR4 recruits either MyD88 or TRIF after endocytosis [30], and induces the production of cytokines and chemokines [53]. Several studies using TLR knockout mice, usually TLR4, demonstrate detrimental effects of TLRs in cerebral ischemia [11, 54]. For example, TLR4-deficient mice do not show increased inflammatory responses compared to wild-type mice post-stroke [54]. The present study showed that SM extract inhibited the up-regulation of hippocampal TLR4 and MyD88 post-BCCAo. Therefore, our results indicate that SM administration may provide protection against chronic cerebral hypoperfusion via MyD88-dependent TLR4 pathways.

We have demonstrated that SM extract protects white matter and hippocampal tissue from damage induced by chronic cerebral hypoperfusion. This is the first report of SM extract in models of chronic BCCAo. However, further studies are needed to characterize the effects of phenolic compounds contained in SM extracts on chronic BCCAo. In spite of several studies about the effect of constituents of SM on learning and memory in dementia and VaD model, few studies have been conducted to investigate the effect of whole SM extract on learning and memory [41, 55]. Therefore, future studies should explore the effects of SM extract on cognitive impairment, which is a common event in patients with VaD.

## Conclusions

In summary, the current study provides data demonstrating the protective effects of SM extract against chronic BCCAo-induced VaD. SM extract decreased the proliferation of microglia and minimized the impairment of MBP in the white matter and hippocampus after BCCAo. SM extract administration also attenuated the release of proinflammatory cytokines and prevented alterations of TLR4 signaling in the hippocampus induced by chronic BCCAo. Taken together, these findings suggest that SM is a potential therapeutic natural medicine for the treatment of VaD.
